# Long-term effectiveness and safety of ustekinumab in bio-naïve and bio-experienced anti-tumor necrosis factor patients with Crohn’s disease: a real-world multicenter Brazilian study

**DOI:** 10.1186/s12876-022-02280-3

**Published:** 2022-04-21

**Authors:** Rogério Serafim Parra, Júlio Maria Fonseca Chebli, Natália Sousa Freitas Queiroz, Aderson Omar Mourão Cintra Damião, Matheus Freitas Cardoso de Azevedo, Liliana Andrade Chebli, Erika Ruback Bertges, Antonio José Tiburcio Alves Junior, Orlando Ambrogini Junior, Bianca Loyo Pona Schiavetti da Silva, Marcio Lubini, Mauro Bafutto, Cristina Flores, Eduardo Garcia Vilela, Sandra Felice Boratto, Newton Luiz Tricarico Gasparetti Junior, Flavio Steinwurz, Nayara Salgado Carvalho, Omar Féres, José Joaquim Ribeiro da Rocha

**Affiliations:** 1 Department of Surgery and Anatomy, Ribeirão Preto Medical School, University of São Paulo, Av. Bandeirantes, 3900, Ribeirão Preto, SP Zip code: 14048-900 Brazil; 2 Federal University of Juiz de Fora, Juiz de Fora, Brazil; 3 Universidade de São Paulo, São Paulo, Brazil; 4 Pontifícia Universidade Católica, Campinas, Brazil; 5 Federal University of São Paulo, São Paulo, Brazil; 6 Prefeitura Municipal de Santos, Santos, Brazil; 7 Passo Fundo University, Passo Fundo, Brazil; 8 Federal University of Goiás, Goiânia, Brazil; 9 Federal University of Rio Grande do Sul, Porto Alegre, Brazil; 10 Federal University of Minas Gerais, Belo Horizonte, Brazil; 11 Centro Universitário Saúde ABC, Santo André, Brazil; 12 Hospital Vivalle Rede D’Or, São José dos Campos, Brazil; 13 Hospital Israelita Albert Einstein, São Paulo, Brazil

**Keywords:** Ustekinumab, Crohn’s disease, Inflammatory bowel disease, Biological therapy

## Abstract

**Background:**

The effectiveness of ustekinumab (UST) in the treatment of Crohn’s disease (CD) has been demonstrated in the pivotal Phase 3 UNITI 1 and 2 and IM-UNITI studies in both anti-TNF-naïve and anti-TNF-exposed patients. Given the selective nature of pivotal trial designs, real-world effectiveness and safety studies are warranted. We report our experience with UST treatment in a large, real-world multicenter cohort of Brazilian patients with CD.

**Methods:**

We performed a retrospective multicenter study including patients with CD, predominantly biologically refractory CD, who received UST. The primary endpoint was the proportion of patients in clinical remission at weeks 8, 24 and 56. Possible predictors of clinical and biological response/remission and safety outcomes were also assessed.

**Results:**

Overall, 245 CD (mean age 39.9 [15–87]) patients were enrolled. Most patients (86.5%) had been previously exposed to biologics. According to nonresponder imputation analysis, the proportions of patients in clinical remission at weeks 8, 24 and 56 were 41.0% (n = 98/239), 64.0% (n = 153/239) and 39.3% (n = 94/239), respectively. A biological response was achieved in 55.4% of patients at week 8, and 59.3% were in steroid-free remission at the end of follow-up. No significant differences in either clinical or biological remission were noted between bio-naïve and bio-experienced patients. Forty-eight patients (19.6%) presented 60 adverse events during the follow-up, of which 8 (13.3%) were considered serious adverse events (3.2% of 245 patients). Overall, a proximal disease location, younger age, perianal involvement, and smoking were associated with lower rates of clinical remission over time.

**Conclusions:**

UST therapy was effective and safe in the long term in this large real-life cohort of Brazilian patients with refractory CD, regardless of previous exposure to other biological agents.

**Supplementary Information:**

The online version contains supplementary material available at 10.1186/s12876-022-02280-3.

## Background

Crohn’s disease (CD) is a chronic, progressive, and idiopathic immune-mediated disorder that can involve any segment of the gastrointestinal tract, likely resulting in negative impacts on the quality of life and work productivity of affected patients [1–6]. The accurate incidence and prevalence of CD in Brazil are still unknown; however, recent regional reports suggest that the incidence of the disease has been increasing over the past years [7].

Anti-tumor necrosis factor (anti-TNF) agents have revolutionized the treatment of CD, allowing control of intestinal inflammation and, consequently, preventing or at least delaying progressive intestinal damage and its harmful consequences, such as the development of strictures, fistulas and abscesses [8, 9]. However, despite its effectiveness, between 10 and 40% of patients with inflammatory bowel disease (IBD) experience primary nonresponse (PNR) depending on the disease type and trial design [10]. In addition, secondary loss of response may occur in approximately 50% of patients who initially respond to anti-TNF therapy during the first year of treatment, leading to the need for intensification or cessation of this therapy [11]. Furthermore, although relatively safe, anti-TNF agents have been associated with an increased risk of drug-related serious and opportunistic infections, malignancies and local or systemic reactions [9, 12, 13].

Hence, new therapies with mechanisms of action other than anti-TNF have been developed and are very welcome to join the therapeutic arsenal targeted at the management of IBD. Ustekinumab (UST) is a fully human monoclonal antibody targeting the p40 subunit of interleukin-12 and interleukin-23 [14]. It has been approved for the treatment of moderately and severely active CD in Brazil since November 2017 [15]. UST is approved in CD for a weight-based intravenous (IV) induction dose (∼6 mg per kg) followed by the first subcutaneous (SC) induction dose at week 8 with a 90 mg injection and subsequent maintenance SC dosing of 90 mg every 8 or 12 weeks at the discretion of the treating clinician.

According to the pivotal Phase 3 UNITI 1 and 2 and IM-UNITI studies, treatment with UST was able to induce and maintain remission in patients with CD and was well tolerated [14, 16, 17]. The effectiveness of this biologic has been established in both anti-TNF-naïve and anti-TNF-exposed patients with higher response rates in the former group of patients. Nonetheless, pivotal trial designs are usually more selective, including a multitude of inclusion and exclusion criteria, so the results are not generalizable for routine clinical practice. Therefore, powerful real-world effectiveness and safety studies are desirable.

Currently, several studies have reported real-world experiences on the effectiveness and safety of UST on standardized dosing regimens [17–22]. These observational studies confirmed the effectiveness of UST in patients with CD but did not include a significant number of bio-naïve patients. Moreover, different demographic, socioeconomic, pharmacogenetics and disease-related factors may exist between Latin American and North American or European populations, which may limit the generalization of these findings. Given that the available data are mainly derived from North American or European populations, real-world studies evaluating the long-term effectiveness and safety of UST for the treatment of CD are lacking in Latin America.

Here, we report our real-world multicenter experience with a standard UST treatment regimen in a large multicenter cohort of Brazilian patients with CD, most of whom were previously exposed to biologics (including anti-TNF agents and/or vedolizumab). We evaluated the long‐term effectiveness and safety of UST in patients with refractory CD as well as predictors of clinical and biological response/remission.

## Methods

### Study design and population

This was an observational, retrospective multicenter study including patients with CD who received the first dose of IV UST. Data from 13 IBD referral centers in Brazil were retrospectively evaluated from November 2017 to November 2019.

The inclusion criteria were moderately to severely active CD at baseline, and the patients must have been treated with at least the initial IV UST (~ 6 mg/kg, weight ranges: < 55 kg: 260 mg, 55–85 kg: 390 mg, > 85 kg: 520 mg). All patients with at least 8 weeks of follow-up from their initial IV dose of UST were included in this study. Moderately to severely active CD was defined as a Harvey-Bradshaw index (HBI) score of 8 or greater or by physician global assessment (PGA) associated with an elevation in the biomarkers (C-reactive protein [CRP] and fecal calprotectin [FC]) in patients who did not have an available HBI in the electronic medical records at baseline.

The exclusion criteria were ulcerative colitis, undetermined colitis, mild disease or in remission at baseline, and incomplete treatment documentation.

All patients received a weight-based IV induction dose (∼6 mg/kg) of UST followed by the first SC induction dose at week 8 with a 90 mg injection. This dose was then followed by a subsequent maintenance SC dosing of 90 mg every 8 or 12 weeks at the discretion of the treating clinician.

### Data collection and ethical approval

Patients were identified at each site through electronic medical record searches. Patient demographic and clinical data were collected by a comprehensive review of their electronic medical records. Data were collected at each site and transferred blinded to the coordinating site (Hospital das Clínicas of the Ribeirão Preto Medical School, University of São Paulo [HCFMRP-USP]). Data collection was performed using a standardized chart review form and prespecified definitions and criteria for coding. The following baseline characteristics were collected: age at inclusion, sex, disease duration, age at diagnosis, disease location and disease behavior according to Montreal classification [23], clinical disease activity, smoking status, steroid dependence, history of perianal CD, previous bowel resection, CRP, FC and hemoglobin levels, extraintestinal manifestations (EIM) or associated immune-mediated inflammatory disease, and previous and current CD treatments (including immunomodulators such as methotrexate [MTX], azathioprine [AZA] or 6-mercaptopurine [6-MP], steroids, anti-TNF therapy or other biologics). In addition, we included information regarding adverse events during the 1st year after UST initiation and drug interruption, surgeries for CD, loss of response, PNR, reasons for drug discontinuation and the need for dose optimization during maintenance therapy with UST.

The study was approved by the ethics committee of the coordinating site (HCFMRP-USP, CAAE: 13436419.7.1001.5440; Ethics Committee Number: 3.335.068/2019). All procedures were conducted in accordance with the 1964 Declaration of Helsinki and its later amendments or comparable ethical standards.

### Definitions and study endpoints

Clinical response was defined as a reduction in HBI of ≥ 3, and clinical remission was defined as an HBI of ≤ 4 in those with HBI ≥ 8 at baseline. In patients without available HBI at baseline, response based on PGA was defined as a ≥ 50% reduction in CD-related symptoms associated with a ≥ 50% reduction in CRP and/or FC levels and remission as complete resolution of all CD-related symptoms associated with biomarker normalization. Clinical assessments for response to therapy were performed based on HBI or PGA and biomarkers at weeks 8, 16, 24 and 56.

Biological response was defined as either a CRP or FC reduction of at least 50% in patients with an elevated CRP or FC at baseline, whereas biological remission was defined as either a CRP or FC level normalization (≤ 5 mg/L and < 250 µg/g, respectively) if these markers were elevated at baseline.

The primary endpoint was the proportion of patients in clinical remission at weeks 8, 24 and 56. The secondary endpoints included the proportion of patients who presented a clinical response at week 8, and a biological response/remission up to week 16 as well as the proportion of patients using baseline steroids who achieved steroid-free remission at the last follow-up, a biological response at week 8, biological remission up to week 16 and a loss of response throughout the treatment.

PNR was defined as the absence of clinical improvement within 16 weeks, leading to drug discontinuation. The loss of response was defined as recurrence of symptoms attributable to CD by the practicing clinician after response to the drug during induction therapy. Steroid-free remission was defined as tapering off steroids completely in patients at baseline, achieving remission and no-repeat steroid prescription within 4 weeks of tapering. Treatment persistence reflects the probability of continuing treatment with UST. It was defined as the duration of time from initiation to discontinuation of UST or switch to another therapy.

### Safety

Safety outcomes were any reported infusion reactions, serious or not serious infections and any serious or not serious adverse events (SAEs). Adverse events (AEs) were defined as serious when they resulted in discontinuation of UST, hospitalization, or death or as considered by the attending physician at the time of its occurrence. Infections were defined as serious when IV antibiotics were required or resulted in discontinuation of UST, hospitalization or death. We collected data on AE throughout treatment with UST as well as reasons for drug discontinuation. Reasons for drug discontinuation included lack of response, surgery for CD, loss of response to UST or serious AEs that would lead to drug discontinuity. Worsening of Crohn’s disease or need for surgery requiring UST withdrawal were not considered AE or SAE. All patients included in the study were used to determine safety outcomes.

### Statistical analysis

Continuous variables were described using the mean with standard deviation (SD) or median and range. Nominal variables were described by frequencies with percentages. The chi-square test and Fisher’s exact test were used for nominal variables, and continuous variables were analyzed using Student’s t-test or Mann–Whitney test. Data were reported using hybrid nonresponder imputation (NRI), last observation carried forward (LOCF) and as-observed analysis. Missing data were imputed as NRI to calculate the proportion of patients in clinical response and remission. The factors related to short- and long-term remission, short-term clinical and biological response and loss of response were analyzed by binary logistic regression analysis, and odds ratios with 95% confidence intervals were calculated. Covariates with a *p* value < 0.20 were selected for the initial model of multivariate analysis. The probability of remission and duration of treatment were estimated using the Kaplan–Meier method, and the differences between the curves were examined using the log-rank test. The period of remission and the duration of treatment (in weeks) were determined from the date of treatment initiation until the date of remission and interruption of medication, respectively, or the data of the last follow-up. Statistical significance was defined as *p* < 0.05. Statistical analysis was performed using SPSS for Windows, version 23.0 (SPSS Inc, Chicago, IL).

## Results

### Baseline characteristics

**Fig. 1:**
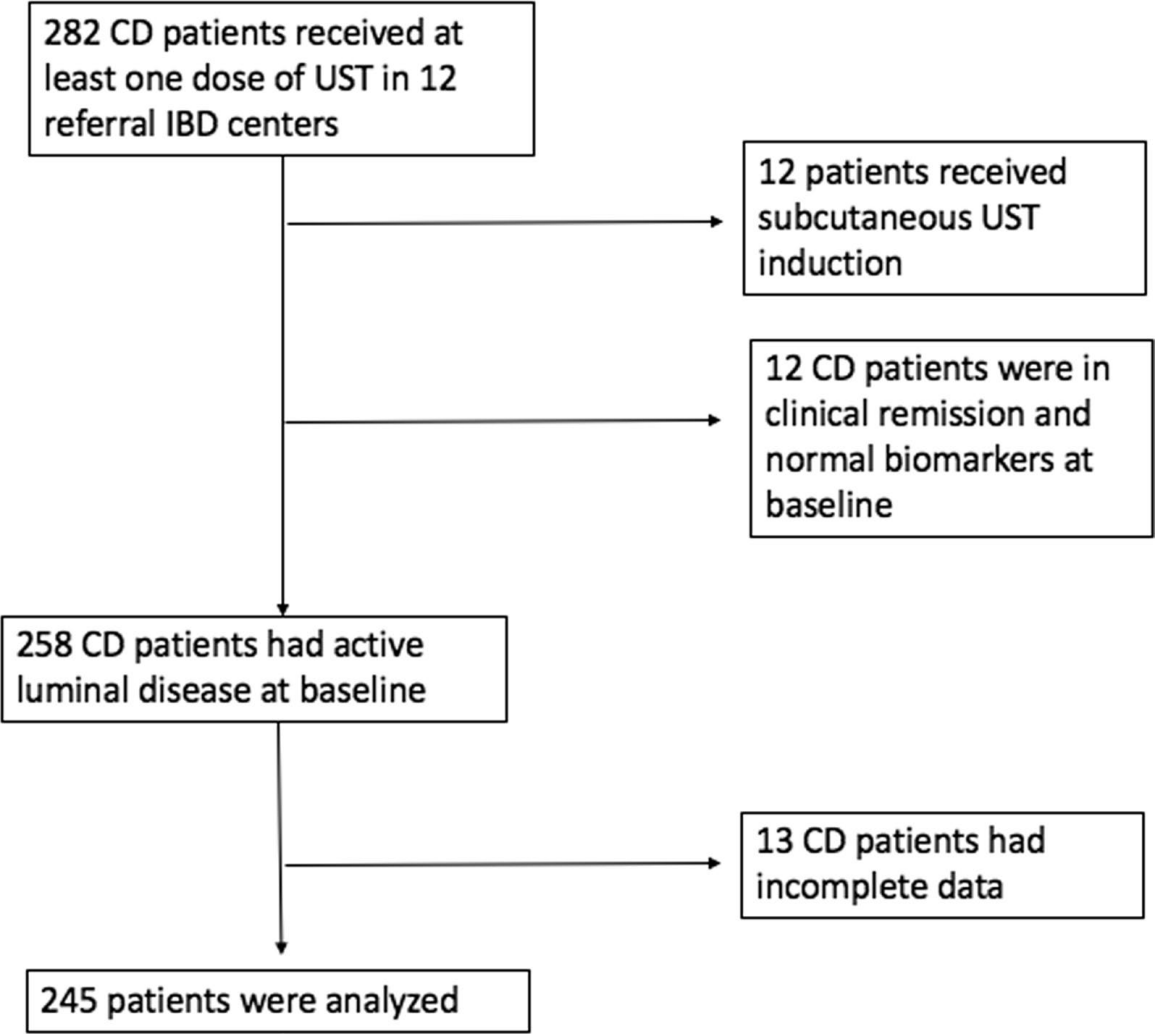
Flow chart of patients with CD using UST. *CD* Crohn’s disease, *UST* ustekinumab, *IBD* inflammatory bowel disease

Overall, 282 patients with CD who had received a single dose of UST up to November 30, 2019, were assessed for eligibility in the study. Among them, 12 (4.2%) patients were in clinical remission and presented normal biomarkers at baseline and were therefore excluded. In addition, 13 (4.6%) patients with incomplete data and 12 (4.2%) patients who had received SC UST induction were excluded. Consequently, 245 (86.9%) subjects were included in this analysis. The flow chart is presented in Fig. 1.

The baseline main characteristics of the participants are described in Table 1. Patients were predominantly female (n = 136, 55.5%) with a median disease duration of 11.0 years (range: 0–38). HBI was available in 170 patients, while the PGA was used for the evaluation of clinical outcomes in 75 patients. Most patients had previous intestinal surgeries (n = 136, 55.5%). The mean age at UST initiation was 39.9 years old (Standard Deviation [SD]: 14.21 years old). In total, 14.7% of patients (n = 36) were currently smokers, 47.4% (n = 116) had anemia and 41.2% (n = 101) had a history of previous or active perianal disease. The majority (87.9%, n = 191) of patients started UST as monotherapy, i.e., without concomitant immunosuppressive medication, such as AZA, 6-MP or MTX. At the beginning of the treatment, 135 patients (60.5%, n = 135/223, missing data in 22 patients) had concomitant use of corticosteroids.

**Table 1:** Baseline clinical and demographic characteristics of 245 patients

Characteristics	Results
Mean age at induction, years (range)	39.9 (15–87)
Gender, n (%)	
Female	136 (55.5)
Male	109 (44.5)
Mean age at diagnosis, years (range)	29.1 (6–78)
Concomitant use of corticosteroids at the beginning of UST treatment^a^, n (%)	135 (60.5)
Montreal classification, n (%)	
Age at diagnosis	
A1 (< 17 years)	44 (18.0)
A2 (17–40 years)	163 (66.5)
A3 (> 40 years)	38 (15.5)
Disease location	
L1 (ileal)	55 (22.4)
L2 (colonic)	35 (14.3)
L3 (ileocolonic)	120 (49.0)
L4 (upper gastrointestinal tract)	35 (14.3)
Behaviour	
B1 (inflammatory)	75 (30.6)
B2/B3 (stenosing/penetrating)	170 (69.4)
Mean disease duration, years (range)	11.0 (0–38)
Disease duration, years, n (%)	
0–2 years	23 (9.4)
3–10 years	111 (45.3)
> 10 years	111 (45.3)
Concomitant use of immunosuppressors^b^, n (%)	54 (22.1)
Previous bowel resection, n (%)	136 (55.5)
Perianal disease^c^, n (%)	101 (41.2)
Active smoking, n (%)	36 (14.7)
Anaemia, n (%)	116 (47.3)
Previous use of biologics, n (%)	212 (86.5)
Number of biologics, n (%)	
0	33 (13.5)
1	71 (29.0)
2	111 (45.3)
3	29 (11.8)
4	1 (0.4)
Previous exposure of biologics, n (%)	
Anti-TNF	182 (74.3)
Infliximab	132 (53.9)
Adalimumab	127 (51.8)
Certolizumab pegol	16 (6.5)
Anti-integrin	27 (11.0)
Vedolizumab	26 (10.6)
Etrolizumab	1 (0.4)

*UST* ustekinumab

^a^There are missing data in 22 patients regarding steroid use at baseline

^b^Thiopurines (azathioprine or 6-mercaptopurine) or methotrexate

^c^Current or prior perianal disease

Most patients (82.4%, n = 202/245) had increased biomarkers (CRP and/or FC) at baseline, whereas 29 (11.8%) patients presented with normal biomarkers (14 patients had missing data). At baseline, the mean HBI was 11 (SD: 3), the median CRP was 10.20 mg/L (range 0.5–125 mg/L), and the median FC was 865.0 mcg/g (range 61–6003 mcg/g). Twenty-three patients (9.4%) had < 2 years of disease duration, 111 (45.3%) had between 3 and 10 years of disease duration, and 111 (45.3%) had > 10 years of disease duration. Most patients had ileocolonic disease (n = 120, 49.0%) and noninflammatory (B2/B3) behavior (n = 170, 69.4%). Most patients were between 17 and 40 years at diagnosis (A2, n = 163, 66.5%). Only 33 patients (13.5%) had never been previously exposed to any biologics (Table 1). Most of them (n = 212, 86.5%) were exposed to at least 1 biologic, including 26 patients (10.6%) previously exposed to vedolizumab. Most patients (n = 142, 58.0%) were previously exposed to two or more biologics. Sixty-one (24.9%) patients presented ≥ 1 EIM mainly with articular involvement.

### UST effectiveness outcomes

**Fig. 2:**
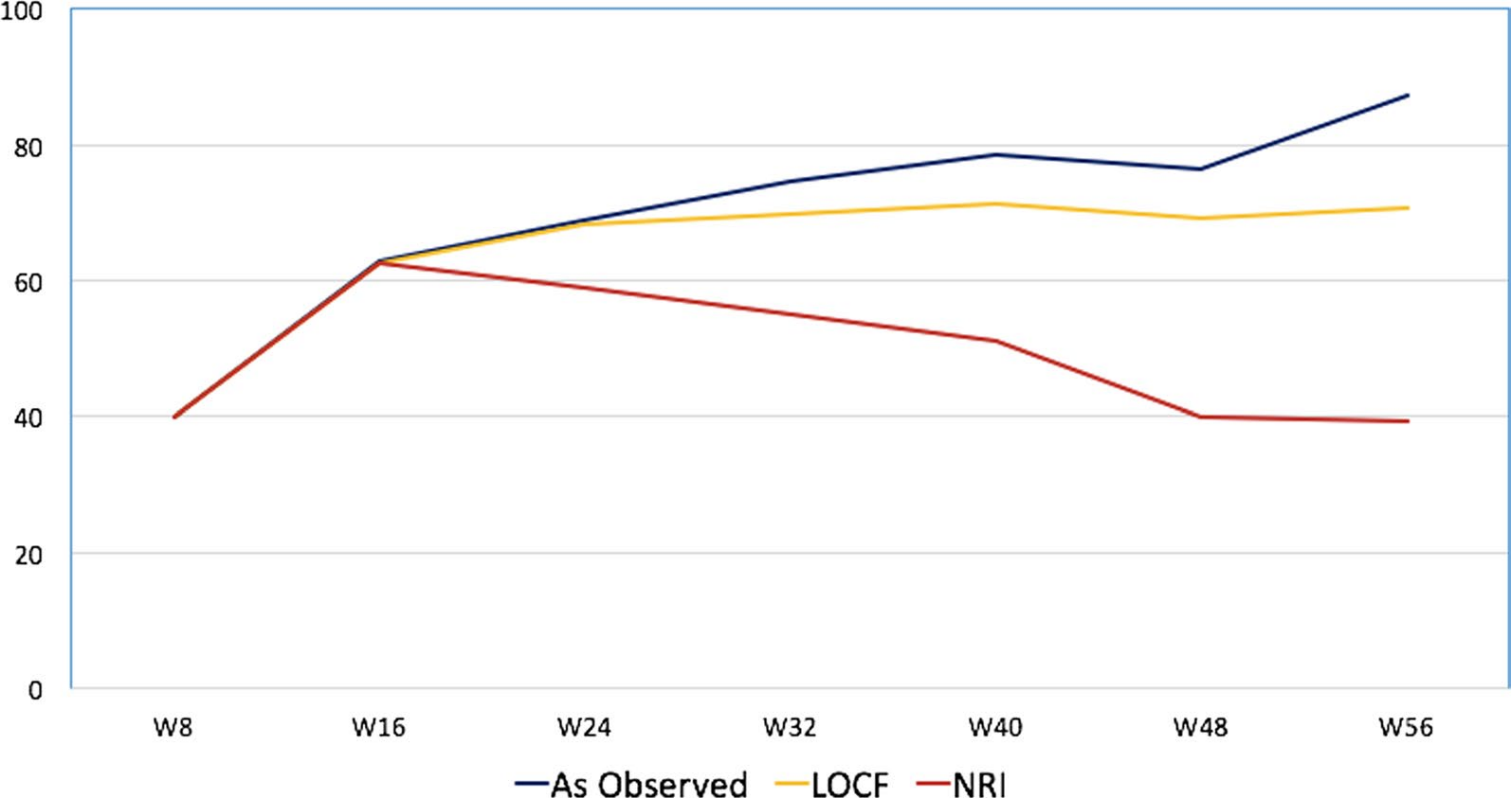
Proportion of patients in clinical remission from weeks 8 to week 56 according to the NRI, LOCF and as-observed analysis. *NRI* nonresponder imputation, *LOCF* last observation carried forward

The proportion of patients in clinical remission during the follow-up period up to week 56 is presented in Fig. 2. In summary, according to NRI analysis, 41.0% (n = 98/239), 60.2% (n = 144/239) and 39.3% (n = 94/239) of patients were in clinical remission at weeks 8, 24 and 56, respectively. According to LOCF analysis, clinical remission rates during the same periods were 41.0% (n = 98/239), 69.9% (n = 167/239) and 72.4% (n = 173/239), respectively. According to the observed analysis, the clinical remission rates were 41.0%, 68.9% and 87.3% at weeks 8, 24 and 56, respectively.

**Fig. 3:**
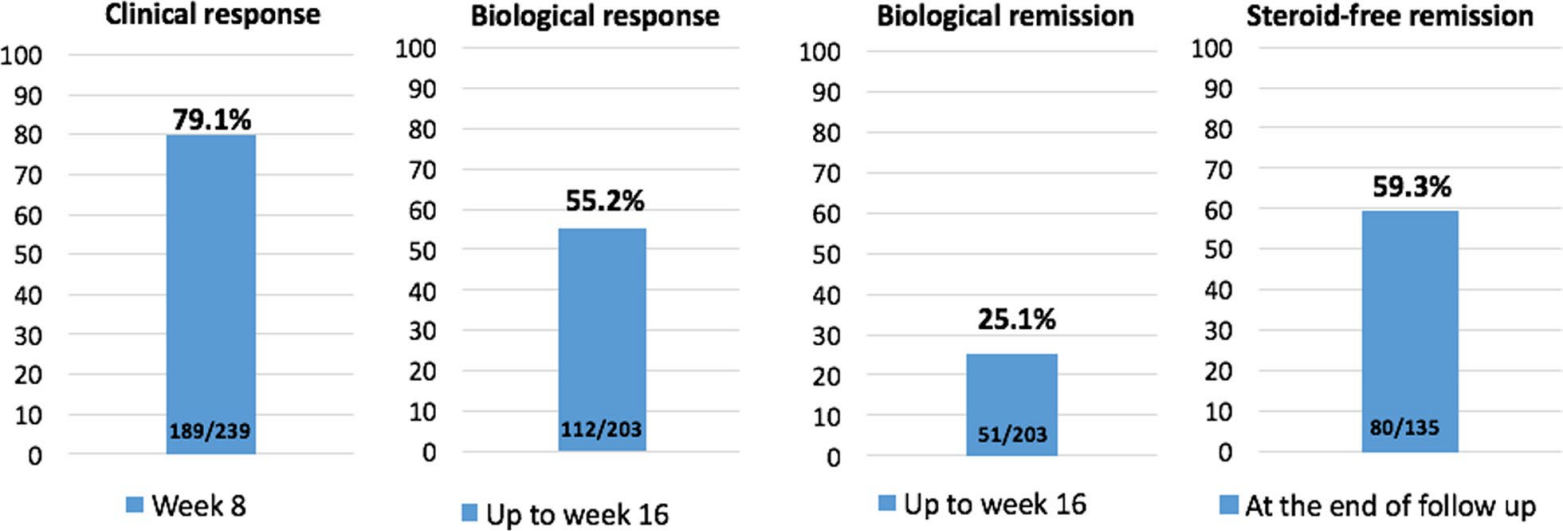
Proportion of patients under UST treatment with clinical response at week 8, biological response/remission up to week 16 and steroid-free remission at the end of follow-up. *NRI* nonresponder imputation, *UST* ustekinumab

The proportion of patients with a clinical response to UST therapy at week 8 was 79.1% (n = 189/239), whereas biological response/remission was achieved in 112 (55.4%, n = 112/202) and 51 patients (51/202, 25.2%) up to week 16, respectively. Notably, steroid-free remission was achieved at the end of the patient’s follow-up in 59.3% of cases (n = 80/135) (Fig. 3).

**Fig. 4:**
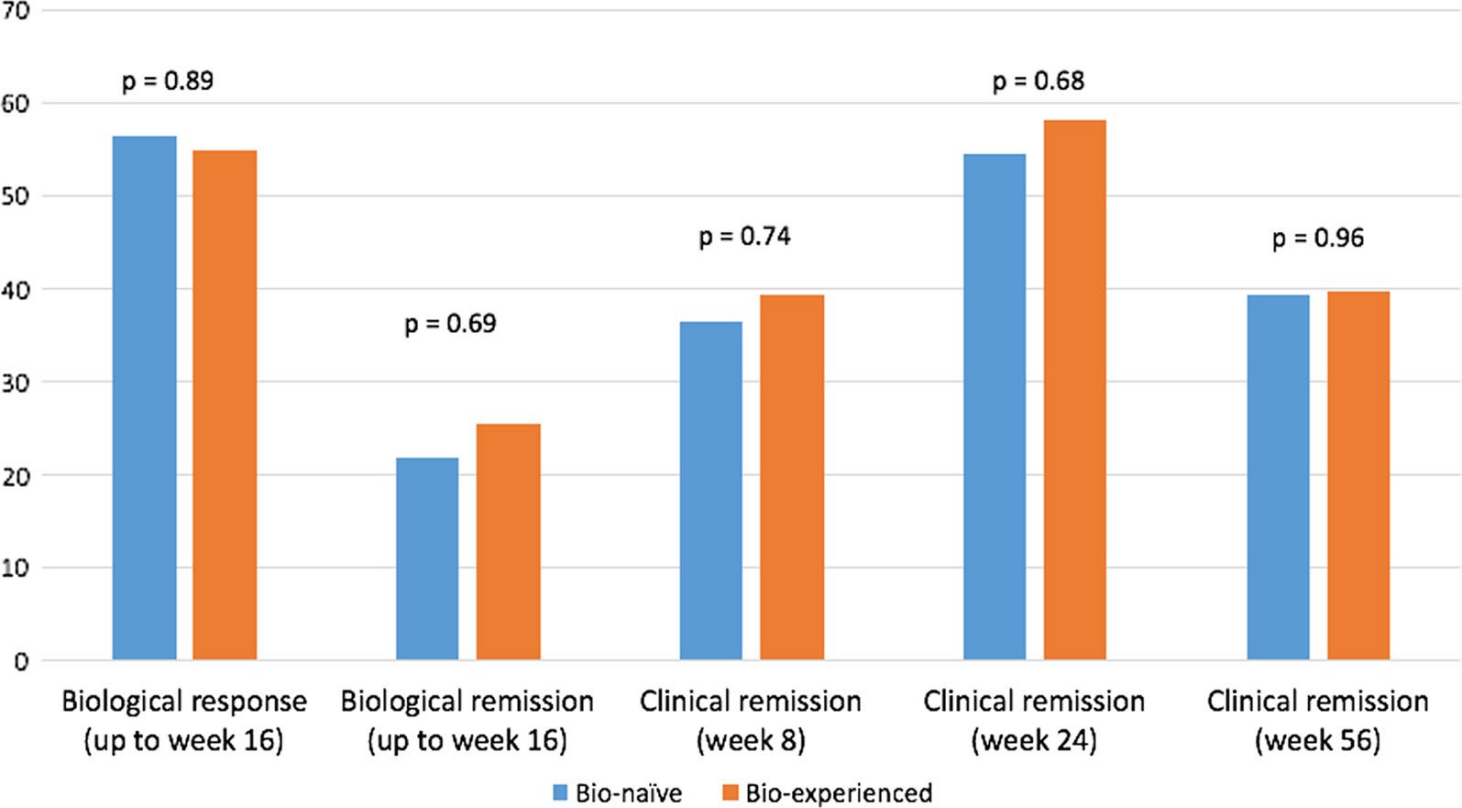
Clinical remission and biological response/remission rates throughout the study comparing bio-naïve and bio-experienced CD patients treated with UST. *NRI* nonresponder imputation, *CD* Crohn’s disease, *UST* ustekinumab

According to NRI analysis, no significant differences were noted between bio-naïve and bio-experienced patients in terms of clinical remission at week 8 (36.4 vs 39.3, respectively; *p* = 0.76), week 24 (54.5 vs 58.2, respectively; *p* = 0.68) and week 56 (39.4 vs 39.8, respectively; *p* = 0.96—Fig. 4). Similarly, bio-naïve individuals did not differ significantly in terms of biological response/remission up to week 16 compared to bio-experienced patients (56.5% vs 55%, *p* = 0.89 and 21.7% vs 25.5%, *p* = 0.69, respectively).

**Fig. 5:**
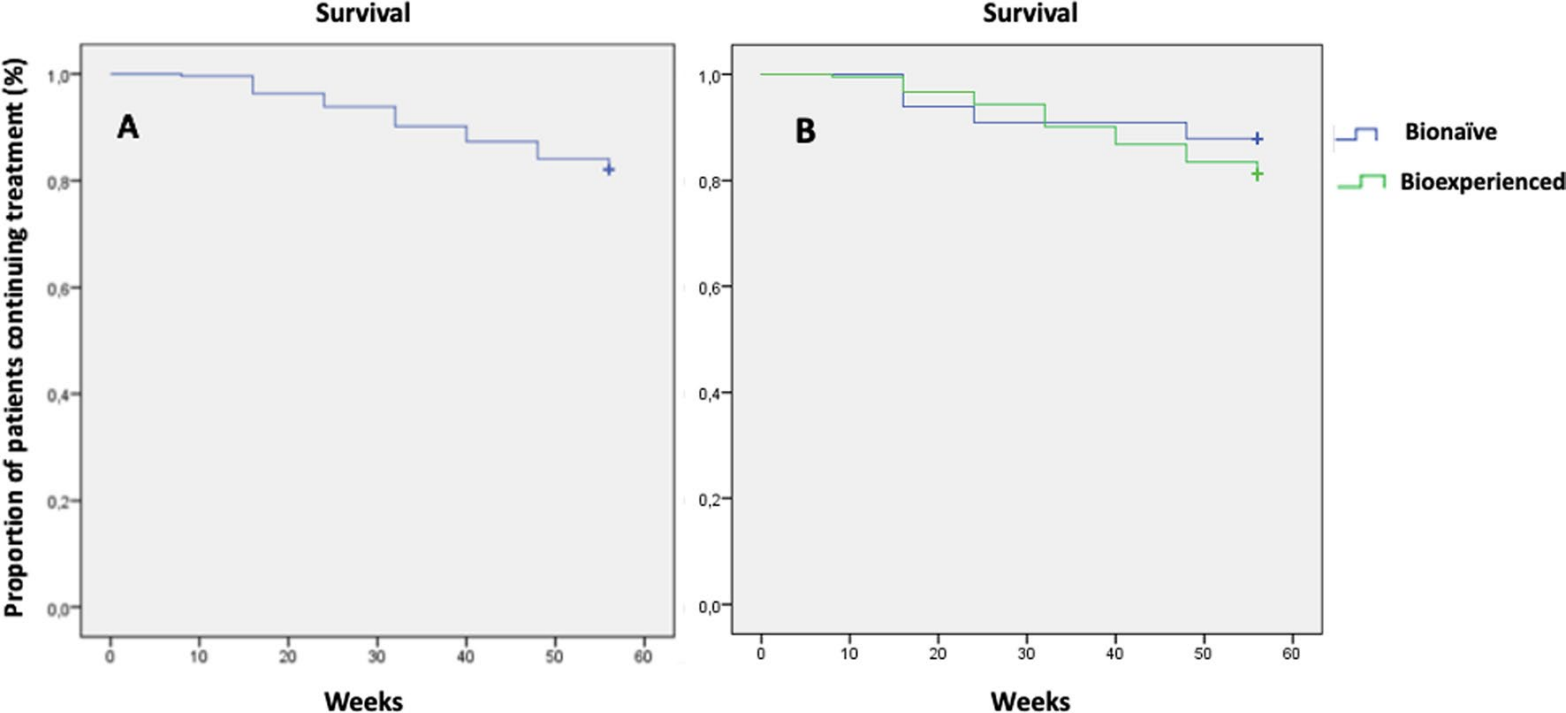
Kaplan–Meier curve for persistence with UST for the whole cohort of patients with CD (**A**), bionaïve (blue line) and bioexperienced (green line) patients (**B**). *CD* Crohn’s disease, *UST* ustekinumab

The probability of continuing treatment with UST assessed by Kaplan–Meier curve analysis is shown in Fig. 5. For the overall cohort, the treatment persistence at the end of follow-up (week 56) was 82% (Fig. 5A). Similar treatment persistence was noted in bio-naïve (82.6%) and bio-experienced patients (82.0%, *p* = 0.90) (Fig. 5B).

The results of the univariate analysis with all variables that have been analyzed in each period are presented in the Additional file 1: Table S1. The following outcomes with their possible predictors that showed a *p* value < 0.20 in the univariate analysis were assessed in a multivariate analysis (logistic regression) for: clinical remission at 8 weeks and 56 weeks, biological remission up to 16 weeks, and loss of response to UST. The results are presented in Table 2. In summary, proximal disease (L4), younger age, perianal disease and combination therapy were associated with lower rates of clinical remission at week 8. Age < 17 years at diagnosis and smoking were associated with lower rates of clinical remission at 56 weeks. Corticosteroid dependency and stricturing/penetrating phenotype were associated with lower rates of biological remission up to week 16, while increased CRP and/or FC at baseline were associated with loss of response to UST during the study.

**Table 2:** Results of the final models of logistic regression analysis for predictors of clinical remission at weeks 8 and 56, biological remission at week 16 and loss of response to ustekinumab

Outcome	Variable	*p* value	Odds ratio	Confidence interval 95%
Clinical remission at week 8	Age	0.004	0.969	0.949–0.990
	Proximal disease (L4)	0.024	1.997	1.095–3.644
	Perianal disease	0.006	0.437	0.241–0.792
	Combotherapy^a^	0.02	0.440	0.220–0.872
Clinical remission at week 56	Age < 17 years (A1)	0.015	5.667	1.395–23.017
	Smoking	0.002	0.110	0.028–0.434
Biological remission at week 16	Steroid dependence	0.009	0.356	0.164–0.769
	B2/B3 behavior	0.003	0.316	0.147–0.681
Loss of response	Increased biomarkers^b^	0.01	2.812	1.276–6201

Only variables in the univariate analysis with a *p* value < 0.20 were considered for the logistic regression model

^a^Combotherapy refers to the concomitant use of ustekinumab with an immunosuppressor (thiopurines or methotrexate)

^b^Increased C-reactive protein (> 5 mg/L) and/or fecal calprotectin (> 250 µg/g) at baseline

### UST dosing

During maintenance, UST 90 mg was subcutaneously administered every 8 weeks in the majority of patients (n = 236, 96.3%). Only 9 patients (3.7%) started the 12-week dose schedule, of which 5 patients were bio-naïve and 4 were previously exposed to biologics (all of them to 2 biologics). Three of these 9 patients (33.3%) needed to shorten the interval to every 8 weeks during the follow-up recapturing the response, and the other 6 patients maintained the dose every 12 weeks up to the end of the follow-up. The need for optimization (to increase the dose from every 8 weeks to every 4 weeks) occurred in 8 patients (3.2%). In 50% of these patients, the optimization resulted in recapture of clinical response, whereas the drug was discontinued due to lack of response, disease progression and/or surgery in the remaining half of patients. None of the patients were reinduced with the IV dose.

### Drug safety

Forty-eight patients (19.6%) presented 60 AEs during the follow-up, of which 8 (13.3%) were considered SAEs (3.2% of 245 patients). Four patients presented bowel perforation (n = 3) or small bowel obstruction (n = 1) during the follow-up (1.6%). Of note, serious infections were reported in only 2 (0.8%) patients, including 1 with hepatic abscess and the other with a gastrointestinal infection. No cases of malignancy or tuberculosis were noted during follow-up. The main mild AEs included cutaneous rash or allergic skin reaction after UST injection (n = 9), and anemia (n = 6), none of which required drug interruption. In addition, various infections (n = 12), including gastrointestinal, upper respiratory tract and cutaneous infections, were also reported. Other less frequent mild AEs included psychological distress symptoms (n = 4), headache (n = 3), and arthralgia (n = 2). The main safety information is presented in Table 3.

**Table 3:** Safety events with ustekinumab treatment during the follow up

Adverse events/serious adverse events	(%)
Any adverse events	48 (19.6)
Most common adverse events^a^	
Diarrhea	7 (2.8)
New perianal fistula	3 (1.2)
Cutaneous rash or allergic skin reaction	9 (3.7)
Anemia	6 (2.4)
Psychological distress symptoms	4 (1.6)
Arthralgia	2 (0.8)
Headache	3 (1.2)
No serious infections	12 (4.9)
Erysipelas	1 (0.4)
Peristomal cutaneous infection	3 (1.2)
Gastrointestinal infection (including *Clostridioides difficile* infection)	6 (2.4)
Upper respiratory tract	2 (0.8)
Lower urinary tract infection	1 (0.4)
Severe adverse events	8 (3.3)
Perianal abscess	2 (0.8)
Bowel perforation	3 (1.2)
Small bowel obstruction	1 (0.4)
Serious infection	2 (0.8)
Gastrointestinal infection	1 (0.4)
Hepatic abscess	1 (0.4)

^a^Other adverse events included 1 case of post-infusion pruritus, 1 case of post-infusion myalgia, 1 case of cavernous sinus thrombosis, 1 case of peristomal ulcer, 1 episode of fever, and 1 episode of asthenia

Drug discontinuation occurred in 44 patients (18%), and the reasons are described in Table 4. Most patients interrupted the medication due to lack of response and disease progression (n = 17, 38.6%) and PNR (n = 12, 27.7%). Two deaths were reported, both in PNR and after treatment withdrawal. The first case was a 21-year-old female patient who had received 1 IV infusion dose of UST; however, due to lack of reimbursement, her treatment was interrupted. She underwent surgery and eventually died due to postoperative complications (dehiscence and sepsis). The second case was a 22-year-old male patient who received 2 doses of UST (IV, followed by week 8 SC) and was considered a PNR with steroid dependence. He received a new induction with infliximab. Due to a lack of response, he underwent surgery and eventually died from postoperative complications (dehiscence and sepsis). However, the cause of death was not considered to be treatment related.

**Table 4:** Reasons for ustekinumab discontinuation throughout the study period

Motive	N (%)
Lack of response/disease progression (with or without surgery)	17 (38.6)
Non-primary response (with or without surgery)	12 (27.3)
Lack of access/reimbursement	7 (15.9)
Pregnancy	6 (13.6)
Depression and treatment abandonment	2 (4.6)
Total	44 (100.0)

## Discussion

In this study, we report our experience with the long-term follow-up of CD patients treated with UST according to the regimens recommended in the UNITI clinical trials in a large, real-world multicenter Brazilian cohort. This cohort of predominantly biologically refractory patients (74.3% anti-TNF, 10.6% vedolizumab) showed a clinical response rate of 77.1% at week 8, and the proportion of patients in clinical remission at weeks 8 and 52 was 40% and 38.3%, respectively, according to the NRI analysis. Biological response was achieved in 55.4% of patients at week 8, and 59.3% were in steroid-free remission at the end of the follow-up period. Overall, proximal disease location, younger age, perianal involvement, combination therapy, stricturing/penetrating behavior, higher HBI at baseline and smoking were associated with lower rates of clinical remission over time.

To our knowledge, this is the first real-world study that evaluated the effectiveness of UST in patients with CD per the currently recommended regimen with IV induction in Latin America. Data from the ENEIDA registry reported rates of clinical remission (defined as HBI < 4) of 47% and 58% at weeks 8 and 14, respectively [21]. In this registry, the proportion of patients with FC and CRP normalization at week 14 was 46% and 35%, respectively. Despite the short-term follow-up (14 weeks), this study showed similar rates of clinical and biological remission following the induction IV dose, as demonstrated in our cohort, and higher rates than those observed in the subgroup of bio-experienced patients in the UNITI‐1 trial (20.9% at week 8) [14]. This discrepancy could be attributed to the different study designs and clinical scoring systems. Moreover, in the real-world scenario, no washout period between one drug and another is required, and there is no restriction for concomitant treatments that could favor a response.

Other real-life cohorts have reported the long-term effectiveness of UST in patients with CD who have been previously exposed to other biologics [17–22, 24, 25]. For instance, in a retrospective, multicenter Belgian cohort, the proportions of patients in clinical remission and steroid-free remission at week 52 were 25.7% and 24.3%, respectively [18]. These lower effectiveness rates could be attributed to the higher previous vedolizumab exposure (69.7%) and higher biological disease activity at baseline (mean CRP: 16.2 mg/L). Interestingly, the rates were quite similar to those observed at weeks 8 and 16, demonstrating sustained remission to UST in those who initially respond to the drug. Accordingly, prospective data from the Dutch Initiative on Crohn and Colitis (ICC) Registry also demonstrated sustained rates of clinical and steroid-free remission over time (40.1% and 39.4% at week 24 and 38.2% and 37.1% at week 52, respectively) [22].

Although no statistically significant difference was observed among the patients receiving UST every 8 or 12 weeks, the q8w maintenance regimen was correlated with a lower discontinuation rate during follow-up. In our study, only 9 (3.6%) patients were treated with the q12w dosing regimen, and 3 of them required interval shortening to q8w during the follow-up recapturing the response. Despite the scarcity of data regarding dose intensification to the q4w interval, our study showed a 50% recapture response rate in the selected group of 8 patients who required dose intensification to the q4w interval. It was recently shown that shortening the UST dose interval is effective and safe and might be beneficial in both clinical and biological indices of disease activity in patients with CD who lost response or did not respond to doses every 8 weeks [26–28].

This study identified the following risk factors associated with lower rates of clinical remission at weeks 8 and 56: younger age, proximal disease, perianal involvement, combination therapy, and smoking. In addition, steroid dependence and stricturing/penetrating behavior were associated with lower rates of biological remission, and increased biomarkers at baseline were associated with a higher risk of loss of response. A large multicenter real-life Spanish cohort identified the number of previous anti-TNF drugs and severe endoscopic activity at baseline as risk factors for inadequate response to treatment. In addition, ileal disease showed a better short-term response [21]. Conversely, the nationwide Belgian cohort found colonic disease to be a positive predictor of response at 1 year [18]. Therefore, one cannot draw definitive conclusions concerning disease location as a predictor of response. As most of the patients included in published real-life cohorts had previously failed one or several other biologics, it is challenging to discuss the influence of previous treatments in the real-world setting.

In the present study, there were no significant differences between bio-naïve and bio-experienced patients in terms of either clinical remission or biological remission. Consistent with this finding, a recent study by Monin et al. [24] evaluated the short- and long-term response to UST in both bio-naïve and bio-failure patients with CD and found that previous biologic failure did not influence the initial response or treatment persistence. In contrast, pivotal studies have previously demonstrated that bio-experienced patients exhibit lower rates of clinical remission compared with those of bio-naïve patients. One possible explanation for this inconsistency could be attributed to the fact that in the real-world setting, clinicians are more prone to initiate UST treatment with the q8w regimen, which could subsequently result in better outcomes independent of previous exposure to biologics. However, this hypothesis should only be considered after replication in other studies and populations.

In our cohort, concomitant treatment with immunomodulators was a consistent risk factor for worse outcomes at week 8. Conversely, pivotal (IMUNITI) [14] and real-world data have clearly demonstrated that the concomitant use of immunomodulators did not significantly contribute to the achievement of higher rates of clinical remission [29, 30]. The findings of our study should be interpreted with caution as the association of combination therapy with lower rates of clinical remission could be the result of unadjusted confounding factors reflecting more severe disease at baseline. Accordingly, steroid dependence at baseline, which may also reflect disease severity, was associated with worse outcomes in our cohort. This finding is consistent with data previously demonstrated in a recent German real-world study that showed steroid dependence as a risk factor inversely associated with remission at week 48 [20].

Given that long-term maintenance treatment without any major event meets the real-life expectations of physicians and patients with CD, drug survival could be interpreted as a surrogate marker of prevention of disability and improved quality of life [31]. Our study showed a drug persistence rate of 82% in 1 year, which is largely consistent with the current literature [32]. Correspondingly, a previous analysis derived from medical and pharmacy claims from US commercial database populations showed that 83.6% of 214 UST-treated patients remained treatment persistent during a 12-month period. Additionally, a Japanese cohort revealed that the cumulative probabilities of maintaining UST treatment at week 52 and week 104 were 89.4% and 81.4%, respectively, which reinforces the higher rate of long-term treatment persistence [33].

The IM-UNITI's 3-year results have confirmed the favorable safety profile of UST treatment over time [34]. Among all UST-treated patients entering the long-term extension study, the number of safety events was not greater than those in the placebo group from week 0 through week 271. In our study, UST treatment was well tolerated, no new safety signals were observed, and treatment discontinuations were mostly attributable to a lack of response/disease progression. Infections occurred in 14 patients during follow-up and were mostly mild and not treatment limiting. The infusion site reaction rate (3.6%) was consistent with those of other SC treatments in IBD, which ranged from 3 to 20% in other reports [35–38], and none resulted in drug discontinuation. The lower rates of mild adverse events could be explained by the retrospective nature of this study as this type of study is prone to underreporting of events without significant clinical implications.

This study has several limitations. First, the study is retrospective in nature, which could result in an underestimation of adverse events, mainly mild adverse events. However, it is essential to note that data were missing in only a few cases. Second, due to the high percentage of previous use of biologics in this cohort, UST was probably maintained in some patients with partial clinical response due to the absence of other therapeutic options. Hence, it was challenging to pinpoint the primary nonresponders. Third, endoscopic data were unavailable to assess mucosal healing, which is a topic that is currently being pursued. Furthermore, although the collection of FC was available in > 50% of the overall cohort, analyses were challenging to manage due to different cutoffs and variability in methods of measurement among 13 centers.

## Conclusion

In conclusion, UST proved to be effective and safe in the real-world long-term treatment of a large cohort of Brazilian patients with refractory CD, regardless of the previous exposure status to other biological agents. A proximal disease location, younger age, perianal involvement, combined therapy, stricturing/penetrating behavior, a higher HBI at baseline and smoking were associated with lower rates of clinical remission throughout the study.

## Supplementary Information

**Additional file 1**. Results of the univariate analysis with all variables that have been analyzed in each period.

## Data Availability

The datasets used and/or analyzed during the current study are available from the corresponding author on reasonable request.

## Abbreviations

CD: Crohn’s disease
Anti-TNF: Anti-tumor necrosis factor
IBD: Inflammatory bowel disease
PNR: Primary nonresponse
UST: Ustekinumab
IV: Intravenous
SC: Subcutaneous
NRI: Nonresponder imputation
HBI: Harvey-Bradshaw index
CRP: C-reactive protein
FC: Fecal calprotectin
PGA: Physician global assessment
MTX: Methotrexate
AZA: Azathioprine
6-MP: 6-Mercaptopurine
SAE: Serious adverse events
AE: Adverse events
SD: Standard deviation
LOCF: Last observation carried forward
