# Supplementary material for: Long-term effectiveness and safety of ustekinumab in bio-naïve and bio-experienced anti-tumor necrosis factor patients with Crohn’s disease: a real-world multicenter Brazilian study
Source: BMC Gastroenterol. 2022 Apr 21;22:199. doi: 10.1186/s12876-022-02280-3 (PMC9027080; doi:10.1186/s12876-022-02280-3)
Supplement: Supplementary file 1 — Additional file 1. Results of the univariate analysis with all variables that have been analyzed in each period. [file 12876_2022_2280_MOESM1_ESM.docx]

Table S1. Results of the univariate analysis with all variables that have been analyzed in each period.

| Outcome | Variable | *P-value* |
| --- | --- | --- |
| Clinical remission at week 8 | Extraintestinal manifestations  Steroid dependence  Proximal disease (L4)  B2/B3 behavior  Perianal disease  Combotherapy*  Age  Age <17 years (A1)  HBI** | 0.107  0.178  0.132  0.128  0.039  0.041  0.120  0.691  0.557 |
| Clinical remission at week 56 | Extraintestinal manifestations  Age <17 years (A1)  Smoking  B2/B3 behavior  HBI** | 0.117  0.167  0.025  0.301  0.236 |
| Biological remission at week 16 | Steroid dependence  Age  Age <40 years (A1/A2)  Age <17 years (A1)  B2/B3 behavior  Anemia at baseline  Perianal disease  Non biological exposure | 0.190  0.510  0.355  0.147  0.016  0.132  0.418  0.352 |
| Loss of response | Increased biomarkers***  Age  Steroid dependence  Clinical remission at week 8  Non biological remission at week 16 | 0.050  0.070  0.520  0.660  0.085 |

*Combotherapy refers to the concomitant use of ustekinumab with an immunosuppressor (thiopurines or methotrexate).

** HBI: Harvey-Bradshaw Index at baseline.

*** Increased C-reactive protein (>5 mg/L) and/or fecal calprotectin (>250 µg/g) at baseline.
